# The Effects of Antioxidant Supplementation on the Pathologic Mechanisms of Metabolic Syndrome and Cardiovascular Disease Development

**DOI:** 10.3390/nu16111641

**Published:** 2024-05-27

**Authors:** Hiva Sharebiani, Mina Mokaram, Melika Mirghani, Bahare Fazeli, Agata Stanek

**Affiliations:** 1 VAS-European Independent Foundation in Angiology/Vascular Medicine, Via GB Grassi 74, 20157 Milan, Italy; hivasharebiani95@gmail.com (H.S.); mirghanimel@gmail.com (M.M.); bahar.fazeli@gmail.com (B.F.); 2 Support Association of Patients of Buerger’s Disease, Buerger’s Disease NGO, Mashhad 9183785195, Iran; minaamokaram@gmail.com; 3 Department of Biochemistry, Faculty of Biological Sciences, Tarbiat Modares University, Tehran 14115-175, Iran; 4 Department of Internal Medicine, Angiology and Physical Medicine, Faculty of Medical Sciences in Zabrze, Medical University of Silesia, Batorego 15 St., 41-902 Bytom, Poland

**Keywords:** metabolic syndrome, obesity, hypertension, oxidative stress, antioxidant supplementation

## Abstract

In people with obesity, diabetes, and hypertension, lipid and glucose metabolism and oxidative stress generation interact. This condition, known as a “metabolic syndrome” (MetS), presents a global challenge and appears to be the underlying mechanism for the development of cardiovascular diseases (CVDs). This review is designed based on evidence indicating the pathogenic mechanisms of MetS. In detail, we will look at the mechanisms of oxidative stress induction in MetS, the effects of elevated oxidative stress levels on the condition’s pathophysiology, and matters related to endothelial function. According to different components of the MetS pathophysiological network, the effects of antioxidants and endothelial dysfunction are reviewed. After considering the strategic role of oxidative stress in the pathophysiology of MetS and its associated CVDs, oxidative stress management by antioxidant supplementation seems an appropriate therapeutic approach.

## 1. Introduction

Metabolic changes in people with obesity, such as elevated levels of glucose, triglycerides (TG), and non-high-density lipoprotein (non-HDL) cholesterol, as well as decreased high-density lipoprotein (HDL) and hypertension, are prevalent aspects relating to cardiovascular diseases (CVDs). Together, these components define metabolic syndrome (MetS), although the new definition of MetS is the presence of obesity and two of the following three criteria: hypertension, impaired glucose metabolism, and atherogenic dyslipidemia [1]. MetS is a risk factor for CVDs and diabetes, doubling the probability of their occurrence. In particular, approximately 25% of the global population suffer from MetS and consequently develop CVDs and diabetes. Therefore, policies for the control and management of MetS are urgently needed to stop the development of these diseases [2,3,4].

There are different mechanisms in the pathophysiology of MetS, including adipose tissue remodeling due to obesity, hyperglycemia, insulin resistance, impaired insulin production, and hypertension [2]. It seems that inflammation and oxidative stress play central roles in these mechanisms; as such, they appear to be appropriate treatment targets for covering all components of this syndrome. Therefore, antioxidants can be suitable supplements for controlling inflammation and oxidative stress, two central mechanisms of MetS [5,6,7,8]. Based on this idea, our objective is to review the pathophysiological mechanisms of MetS, introducing oxidative stress as its central mechanism, and the possible benefits of antioxidant therapy.

In this review, we focus on the evidenced mechanisms of MetS. Table 1 shows the level of evidence of the studies included in this review.

When considering antioxidant therapy for a complex pathological condition like MetS, it is necessary to understand its pathophysiological mechanisms, especially the role of oxidative stress. These mechanisms include adipose tissue remodeling due to obesity, hyperglycemia, insulin resistance, impaired insulin production, and hypertension [2], all of which are reviewed in Section 2. In the next section, the role of each MetS component in the development of CVD is demonstrated. In Section 4, their roles in inducing oxidative stress, as well as the effects of oxidative stress on the condition’s pathophysiology and CVD development, are discussed. Since inflammation and oxidative stress appear to play central roles in these mechanisms, they appear to be appropriate treatment targets for covering all components of this syndrome. Since antioxidants can be suitable supplements for controlling inflammation and oxidative stress, the central mechanisms of MetS, Section 5 is dedicated to antioxidant therapy [5,6,7,8]. In order to consider the role of environmental factors of MetS incidence, the syndrome’s epigenetic changes are reviewed in Section 6. Section 7 demonstrates the MetS pathophysiological network, as well as the challenges in its management, by discussing the pathophysiological mechanisms, especially those related to oxidative stress; introducing oxidative stress as the central mechanism of MetS; and exploring the possible benefits of antioxidant therapy.

## 2. Pathophysiological Mechanisms of Metabolic Syndrome

**Figure 1:**
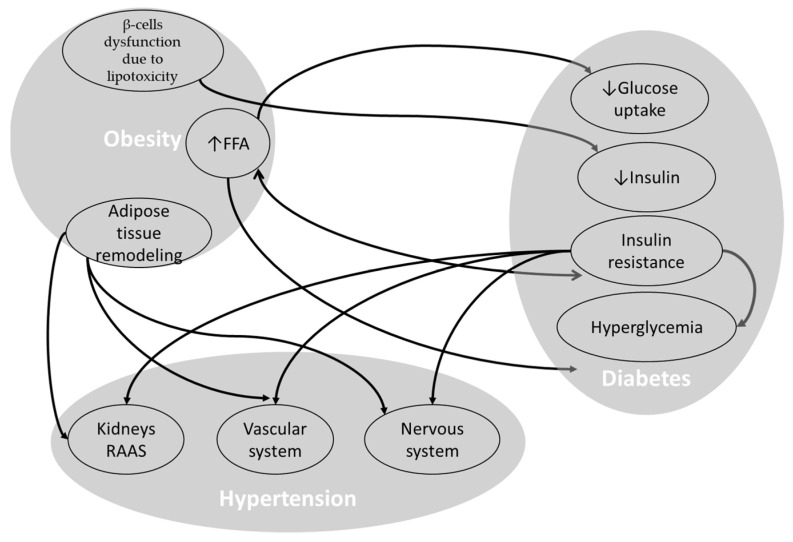
Interactions of the MetS components, obesity, diabetes, and hypertension. FFA: free fatty acid; RAAS: renin–angiotensin–aldosterone system; ↑: increase; ↓: decrease.

In order to review the pathophysiology of MetS, there are three main considerations: (i) obesity, adipose tissue remodeling, and dyslipidemia; (ii) diabetes, insulin resistance, and hyperglycemia; and (iii) hypertension. For each of these, the group of mechanisms and their interactions are discussed. Figure 1 shows the interactions of MetS components. According to this classification, the hub or strategic point in this complex network could be found for targeted therapy.

### 2.1. Obesity, Adipose Tissue Remodeling, and Dyslipidemia

Obesity is a global challenge characterized by the excessive accumulation of body fat and the expansion and remodeling of adipose tissue. In those with obesity, the high metabolic activity of the accumulated visceral fat releases free fatty acids, which can lead to hyperinsulinemia, dyslipidemia, inflammation, and atherosclerosis. The infiltration of immune cells, elevated levels of leptin, resistin, plasminogen activator inhibitor-1 (PAI-1), and pro-inflammatory adipokines, and the decrease in the thermogenesis of adiponectin and brown adipose tissue (BAT) are the main incidences of adipose tissue remodeling in people with obesity. These changes can induce both inflammation and oxidative stress [6,8,9,10]. Diet, host genetics, gut microbiota, and other factors related to obesity participate in inducing the adipose tissue inflammation that results in metabolic disorders [11]. Even post-translational alterations like splicing are important in adipogenesis and thermogenesis [12].

In people with obesity, increasing the production of very low-density lipoproteins (VLDL) and HDL catabolism, along with a simultaneous reduction in the catabolism of particles containing apo-B, lead to dyslipidemia. In people with obesity, elevated levels of FFA released from adipose tissue can inhibit glucose uptake by skeletal muscle cells, which is mediated by insulin. This leads to a reduction in insulin sensitivity in muscles and, consequently, the pancreas tries to compensate for it and maintain glucose tolerance by overproducing insulin. Chronic hyperinsulinemia reduces insulin receptors, desensitizes post-receptor mechanisms, and, finally, leads to insulin resistance. Despite the elevation in FFA, its oxidation capacity in muscle cells is decreased, increasing the accumulation of fatty acyl-CoA and TG in cells [12,13]. Furthermore, the decrease in adiponectin during obesity contributes to insulin resistance, due to the insulin-sensitizing role of adiponectin [13]. Additionally, there appears to be a mutual interaction between dyslipidemia and both oxidative stress and inflammation [14,15,16]. Elevated levels of malondialdehyde (MDA) in hyperlipidemic individuals indicate that oxidative stress is one of the important consequences of hyperlipidemia [15].

### 2.2. Diabetes, Insulin Resistance, and Hyperglycemia

Type 2 diabetes (T2D), the most prevalent type of diabetes and a growing global challenge that affects about half a billion people around the world, is associated with MetS. The chance of developing T2D in people with MetS is five times higher than in nondiabetic individuals [4]. Furthermore, pathophysiological changes in people with T2D can lead to the development of MetS. In addition to MetS, obesity, dyslipidemia, and oxidative stress contribute to the development of T2D [16,17].

Diabetes has a complex and multifactorial pathophysiology that includes insulin resistance, decreased pancreatic insulin secretion, and hormonal dysregulation in the enterohypothalamic and enteroinsular axes. These factors are affected by genetics and the environment [4]. On the one hand, as mentioned below, dyslipidemia is one of the underlying mechanisms that induces insulin resistance. Glucose uptake, insulin signaling, glycogen synthesis, and gluconeogenesis are the mechanisms involved in the pathophysiology of diabetes that are affected by TG metabolites [4]. On the other hand, insulin resistance induces dyslipidemia by affecting the low-density lipoprotein receptor (LDL) and consequently removing LDL, decreasing lipoprotein lipase (LPL) activity, and overactivating liver lipase (HL). HL hydrolyzes TG-rich LDL and TG-rich HDL into dense LDL and dense HDL, respectively. The former cannot be efficiently removed from the plasma, while the latter is cleared quickly [18].

The inhibition of lipolysis by insulin in adipose tissue is one of the other mechanisms that is impaired due to insulin resistance; it also leads to an increase in circulating FFA and worsened insulin resistance [3]. Elevated FFA reduces glucose uptake by decreasing the presence of glucose transporter-4 (GLUT-4) on the surface of cells and simultaneously up-regulates gluconeogenesis and lipogenesis in the liver [3,19]. Elevated FFA promotes both inflammation and oxidative stress by activating the nuclear factor-κB (NF-κB) signaling pathway, inducing mitochondrial dysfunction, and altering the endogenous antioxidant system [16]. High levels of lipids in β-cells can induce lipotoxicity due to the excessive oxidation of fatty acids and overproduction of reactive oxygen species (ROS). This toxicity leads to β-cell dysfunction and impaired insulin secretion [3,16]. The activation of Toll-like receptor 4 (TLR4) by FFA is another trigger of insulin resistance [17].

In addition to changes in lipid metabolism, dyslipidemia, and visceral obesity, insulin resistance leads to hyperglycemia, hypertension, inflammation, oxidative stress, thrombosis, and endothelial dysfunction, which are the underlying mechanisms in the development of MetS, non-alcoholic fatty liver disease (NAFLD), and T2D [3,7,19]. Obesity and MetS generate oxidative stress due to increased levels of glucose, FFA, and insulin, which increase hyperinsulinemia and insulin resistance [16]. Similarly, diabetes can induce high levels of oxidative stress due to elevated levels of glucose, glucose autoxidation, the formation of advanced glycation end products (AGEs), the polyol pathway, and protein kinase C (PKC) [16,20,21].

Insulin resistance, decreased glucose uptake, the up-regulation of gluconeogenesis, and glycogenolysis induce hyperglycemia in patients with T2D [17,18]. Hyperglycemia can cause glucose toxicity, oxidative stress, inflammation, dyslipidemia, hypertension, and endothelial dysfunction. The production of MDA due to lipid peroxidation is one of the indicators of hyperglycemic ROS generation [21]. Hyperglycemia’s role in inducing ROS and inflammatory processes leads to insulin resistance, β-cell dysfunction, and, consequently, further hyperglycemia [16,17]. Furthermore, both hyperglycemia and lipid peroxidation can affect total antioxidant capacity [21].

### 2.3. Hypertension

Hypertension, a powerful risk factor for CVD development, is responsible for about one third of global mortality [21]. Both obesity-related mechanisms, such as adipose tissue remodeling and dyslipidemia, and diabetes-related mechanisms, such as hyperinsulinemia and insulin resistance, play roles in the pathophysiology of hypertension in people with MetS [22].

There are several mechanisms involved in the obesity–hypertension phenotype. Affecting vascular tonicity, the overactivation of both the sympathetic system and the renin–angiotensin–aldosterone system (RAAS), as well as physical stress on the kidneys, are the main mechanisms. Elevated arterial tone occurs due to high levels of FFA increasing α-adrenergic vascular sensitivity. Furthermore, in people with obesity, hyperlipidemia, elevated levels of circulating leptin, and baroreflex sensitivity dysfunction result in the overactivation of the sympathetic nervous system [10,22,23]. As the main regulator of blood pressure, RAAS is affected by obesity. On the one hand, adipose tissue functions as a source of renin, angiotensin, and aldosterone, enhancing their levels in the circulatory system. However, RAAS receptors are located in this type of tissue; its interaction with glucocorticoids results in increased RAAS activity and, as a result, hypertension [10]. Enhancing renal tubular re-absorption, thus promoting sodium retention, is reported to be one of the initial effects of obesity on the renal system. Therefore, obesity-related hypertension involves salt sensitivity and volume expansion. Furthermore, kidney compression due to excessive adipose tissue in people with obesity affects both the vascular and tubular systems, leading to sodium re-absorption and the overactivation of RAAS [22,23]. Studies show that there is a direct correlation between the elevated fractional absorption of sodium in the proximal tubule and both body mass index (BMI) and blood pressure. Moreover, an improved BMI can lead to the down-regulation of RAAS [22].

Furthermore, the interaction between insulin and the vascular, renal, and nervous systems indicates the pivotal roles of hyperinsulinemia and insulin resistance in hypertension. Arterial dysfunction and vasoconstriction have been reported to be significant consequences of chronic hyperinsulinemia. Additionally, insulin promotes sodium re-absorption and sodium retention via its direct effect on the kidneys [10,22], although its indirect effects are applicable through the overactivation of the sympathetic nervous system [22,23]. Furthermore, excessive fructose consumption can stimulate salt absorption in the small intestine and kidney tubules, promoting hypertension [22]. There is a difference between patients with hypertension alone and those with both hypertension and MetS. Due to the mechanisms involved in the formation of MetS-related hypertension, there is a greater chance of target organ damage, such as left ventricular hypertrophy, aortic stiffness, and microalbuminuria [24].

## 3. The Role of Metabolic Syndrome in the Development of CVDs

Each component of MetS affects the vascular system in a particular way and there are overlapping effects and interactions between them. All these components, including obesity, diabetes, and hypertension, are associated with CVD and its related mortality.

### 3.1. Obesity, Adipose Tissue Remodeling, and Dyslipidemia

As a risk factor for CVD, obesity affects the vascular system by inducing inflammation, oxidative stress, vascular aging, and endothelial dysfunction [6]. The reduced bioavailability of nitric oxide (NO), vascular remodeling and stiffness, the adhesion of monocytes to the endothelium, macrophage polarization, and endothelial permeability are the main effects of obesity on the vascular system [25].

In obesity, macrophage infiltration, the activation of Toll-like receptors and Jun *N*-terminal kinase (JNK), the inhibition of NF-B kinase (IKK) and protein NF-κB kinase (PKR), elevated levels of pro-inflammatory adipokines and cytokines, and reductions in NO, adiponectin, IL-10, and PGL2 (prostacyclin) cause both endothelial dysfunction and metabolic disorders [24]. In particular, in MetS, alterations in the levels of leptin, resistin, tumor necrosis factor-α (TNF-α), IL-6, angiotensin II, endothelin-1 (ET-1), and adiponectin lead to endothelial dysfunction and vasoconstriction [25]. In both obesity and MetS, there are elevated levels of factors related to vascular endothelial integrity, including a thrombotic tendency mediated by vascular endothelial growth factor-1 (VEGF-1) and PAI-1, which can lead to vascular dysfunction and thrombosis [24].

A reduction in anti-atherogenic particles and an increase in atherogenic particles are the mechanisms through which dyslipidemia causes pathological changes in the vascular system. HDL functions as an anti-atherogenic, anti-inflammatory, and antithrombotic factor that participates in improving endothelial function and repair. Additionally, HDL removes cholesterol from cells and inhibits LDL oxidation. The atherogenicity of LDL particles is associated with their low affinity for the LDL receptor, their susceptibility to oxidation, and their down-regulation of NO synthase, as well as their increased binding to heparan sulphate proteoglycans in the arterial wall matrix [18].

Perivascular adipose tissue (PVAT) plays a significant role in the regulation of vascular tone, endothelial function, and blood pressure. Inflammation due to the presence of pro-inflammatory adipokines like TNF-α, monocyte chemotactic protein-1 (MCP-1), IL-6, and IL-8 results in vascular insulin resistance, vasoconstriction, and vascular stiffness [25]. Obesity-induced PVAT dysfunction leads to a reduction in NO bioavailability, inflammation, and an increased activation of RAAS [26].

### 3.2. Diabetes, Insulin Resistance, and Hyperglycemia

Diabetes is a well-known risk factor for CVDs, inducing microvascular and macrovascular complications [27], and insulin metabolism and endothelial function exhibit interactions. Insulin resistance can alter endothelial signaling and cause vasoconstriction, prothrombotic status, and endothelial dysfunction. Furthermore, elevated levels of inflammatory mediators and oxidative stress are other consequences induced by insulin resistance in endothelial cells [17]. As one of the main vascular consequences of insulin resistance, vasoconstriction can occur directly through the diminished insulin vasodilatory effect and indirectly by stimulating the RAAS and sympathetic system [3]. One of the main mechanisms controlling vascular tone is NO production. A reduction in NO bioavailability is the hallmark of endothelial dysfunction; it occurs during the activation of the mitogen-activated protein kinase (MAPK) signaling pathway due to hyperinsulinemia and RAAS overactivity [17]. Increased serum viscosity during insulin resistance makes patients with T2D susceptible to thrombotic events [3]. Insulin resistance can also change the gene expression of the estrogen receptor, leading to the formation of atherosclerosis plaques [17].

Glucose-induced endothelial dysfunction involves different mechanisms. A high level of oxidative stress, as a consequence of a high glucose metabolic rate in endothelial cells during hyperglycemia, is one of the mechanisms of glucose-induced endothelial dysfunction. AGEs are other sources that can damage endothelial cells by modifying plasma, extracellular matrix, and intracellular proteins. The activation of the PKC signaling pathway caused by hyperglycemia leads to a reduction in endothelial nitric oxide synthase (eNOS) and promotes oxidative stress through increased nicotinamide adenine dinucleotide phosphate (NADPH) oxidase (NOX). Glucose-induced endothelial dysfunction can also occur during hyperglycemia due to protein O-GlcNAcylation and an increased production of UDP-GlcNAc [28]. Furthermore, glucose can affect vascular tone due to changes in endothelium-dependent vasodilation. Therefore, hyperglycemia can reduce NO levels and induce vasoconstriction in both the macro- and microcirculation [25].

### 3.3. Hypertension

There is a mutual interaction between hypertension and endothelial dysfunction. In particular, vascular dysfunction caused by dyslipidemia, adipose tissue remodeling, insulin metabolism dysfunction, and hyperglycemia leads to hypertension. The underlying mechanisms of this hypertension are vasoconstriction, decreased vasodilatory mechanisms, elevated intravascular fluid, and sympathetic overactivity [29]. On the other hand, high blood pressure induces vascular remodeling [30].

Hypertension is a significant risk factor for CVD due to its influence on vasculature, resulting in vascular abnormalities and procoagulant states [31]. The decrease in outer diameters and lumen, the elevated media/lumen ratio, and the thickening of the media are structural changes that hypertension indues in small arteries. Apoptosis, fibrosis, the growth and rearrangement of smooth muscle cells, matrix accumulation, inflammation, and oxidative stress are the main mechanisms involved in this vascular remodeling [30]. In hypertensive individuals, changes occur in the function of the arterial wall’s vascular endothelium. Endothelial cell dysfunction includes enhanced procontractile factors, such as ET-1, angiotensin II, and prostaglandins, and the reduced secretion of pro-relaxant factors such as NO, PGI2, and endothelium-derived hyperpolarizing factor (EDHF). Increased endothelium permeability, barrier dysfunction, and endothelial cell metabolic dysfunction are other changes in endothelium function that are seen during hypertension. Furthermore, inflammation, fibrosis, and calcification occur in tunica media due to the increase in the damage-associated molecular pattern (DAMP) and matrix vesicles in vascular smooth muscle cells. The secretion of matrix metallopeptidase 9 (MMP-9), pro-inflammatory cytokines, and fibrocyte chemokines leads to the deposition and inflammation of the connective tissue matrix in tunica media. Collagen deposition and inflammation in adventitia are also induced by high levels of cytokines, chemokines, and growth factors released by fibroblasts. Furthermore, PVAT remodeling leads to inflammation, NO bioavailability, and the overactivation of RAAS. The elevated growth factors released by pericytes in adventitia contribute to angiogenesis, vasodilation, and inflammation [24,28,30].

These pathophysiological changes in the vascular system can contribute to the development of CVD. Studies have demonstrated that there is a J-shaped association (nonlinear) between high blood pressure and vascular disease [32]. The risk of peripheral arterial disease (PAD) in patients with hypertension is 2.5- to 4-fold higher than in healthy individuals [32]. Controlling blood pressure in patients with PAD is a critical challenge because the ability to adequately perfusing the coronary and peripheral arteries is blood pressure-dependent. Therefore, lowering blood pressure below a critical value can lead to cardiovascular events [33,34].

## 4. Oxidative Stress as a Strategic Mechanism Involved in the Pathophysiology of MetS

**Figure 2:**
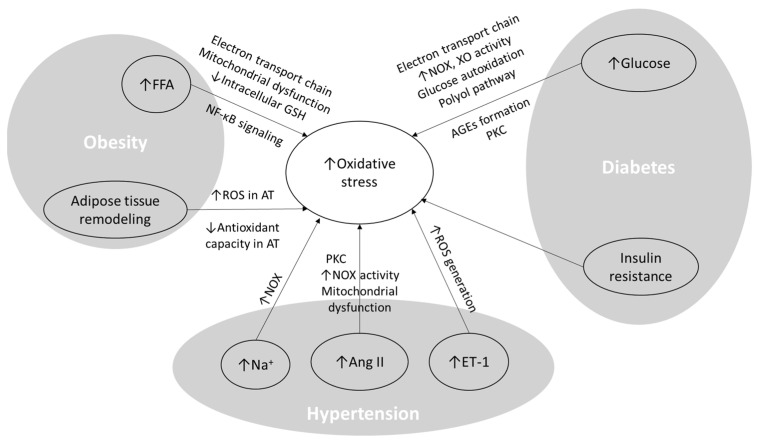
The role of obesity, diabetes, and hypertension in inducing oxidative stress. FFA: free fatty acid; Ang II: angiotensin II; ET-1: endothelin-1; GSH: glutathione; AT: adipose tissue; ROS: reactive oxygen species; PKC: protein kinase C; NOX: NADPH oxidase; XO: xanthine oxidase; NF-κB: nuclear factor-κB; ↑: increase; ↓: decrease.

All components of MetS, including obesity, adipose tissue remodeling and dyslipidemia, hyperglycemia, insulin resistance and hyperinsulinemia, and hypertension, participate in the production of oxidants and the activation of oxidative stress pathways (Figure 2). Therefore, oxidative stress seems to be a strategic point in MetS pathophysiology. In this section, the role of each component in the generation of oxidative stress is first demonstrated. The effects of elevated oxidative stress on MetS components and the vascular system are described below.

### 4.1. The Role of MetS Pathophysiological Components in Inducing Oxidative Stress

High levels of oxidative stress result from two concurrent phenomena: (i) the excessive generation of oxidants and (ii) impaired antioxidant systems. There are different mechanisms in MetS that lead to excessive ROS generation, including high levels of fatty acid (FA) and glucose metabolism, MetS salt-sensitive hypertension, obesity-related adipose tissue remodeling, hyperinsulinemia, and inflammation [8,16,22,35]. On the other hand, in MetS, antioxidant activity is reduced. The activities of superoxide dismutase (SOD), catalases (CAT), and glutathione peroxidases (GPx) have been reported to be inversely related to BMI [8].

The main sources of ROS generation are the electron transport chain in the mitochondrial, peroxisome, and cytochrome P450 systems [16], although there are other sources, such as xanthine oxidase (XO), lipoxygenase, NOX, cyclooxygenases, myeloperoxidase, uncoupled eNOS, and pro-oxidant heme molecules [35]. The antioxidant system divides enzymatic and non-enzymatic antioxidants. As the first line of defense, enzymatic antioxidants fulfill their antioxidative role by preventing or suppressing ROS generation. These antioxidants include SOD, CAT, GPx, glutathione reductases (GR), glutathione-S-transferases, thioredoxin reductase (TrxR), peroxiredoxins, and reduced NADPH: ubiquinone oxidoreductase. The second line of defense includes vitamins C and E, coenzyme Q, and glutathione (GSH), which are non-enzymatic antioxidants that participate in neutralizing free radicals [8].

Overnutrition is the first component of MetS involved in the overproduction of ROS. Excessive FA and glucose metabolism leads to an increase in NADH and FADH2 production in the electron transport chain [16,36]. Elevated FFA increases β-oxidation in mitochondria and results in a higher NADH/NAD^+^ ratio and AGE, as well as the inhibition of eNOS and NO levels due to PKC overactivation [37]. Furthermore, overnutrition can activate NOX as one of the main sources of ROS generation in cells [35,38]. In particular, by NOX activation, hyperglycemia elevates ROS generation through the up-regulation of PKC and the Ca^2+^/calmodulin-dependent protein kinase and AGE/RAGE signaling pathways [35].

The second component of MetS that contributes to elevated levels of oxidative stress is obesity and the remodeling of adipose tissue. ROS are overexpressed in adipose tissue, and this is mainly induced by NOX and a decreased antioxidant capacity. Elevated FA is associated with ROS overproduction due to mitochondrial dysfunction, as well as a decrease in intracellular glutathione. Furthermore, FA can act as a pro-inflammatory factor that activates NF-κB [16].

We often see MetS begins after the age of 45 in men and 55 in women. At these ages, the major source of energy production is FA, which can lead to elevated ROS production, and accelerate the aging process. Moreover, changes in life style at this age, like excessive food consumption and fewer physical activities, can exacerbate these issues [39].

Together, hyperinsulinemia, diabetes, and insulin resistance can be considered the third component of MetS, inducing oxidative stress due to mechanisms such as stimulating NADPH-dependent H_2_O_2_ production or the selective enhancement of XO [37,40,41,42]. In patients with T2D and MetS, elevated XO activity has been reported [42]. Furthermore, there are other mechanisms related to diabetes that lead to ROS generation, such as hexosamine pathways, glucose autoxidation, polyol pathway, PKCβ1/2 kinase, and AGEs [16]. Furthermore, there is evidence that insulin resistance can induce oxidative stress. [8,29]. Due to interference with insulin signaling, high levels of oxidative stress in adipocytes contribute to insulin resistance [8].

The last major component in generating oxidative stress in MetS is salt-sensitive hypertension. Sodium retention and salt sensitivity in MetS patients result in high sodium levels, which can up-regulate the expression of NOX and SOD and induce elevated ROS levels [22]. Furthermore, high blood pressure can directly or indirectly increase oxidative stress by affecting vasoactive peptides such as angiotensin II and ET-1 [30]. The activation of NOX through angiotensin II-mediated PKC can induce mitochondrial dysfunction in endothelial cells and cardiomyocytes [35].

As a common factor in all the mentioned mechanisms, such as obesity, adipose tissue remodeling, hyperglycemia, the accumulation of FFA and AGE, and systemic insulin resistance, inflammation plays a central role in inducing oxidative stress [35].

### 4.2. Effects of Oxidative Stress on the Pathophysiology of MetS and the Development of CVDs

**Figure 3:**
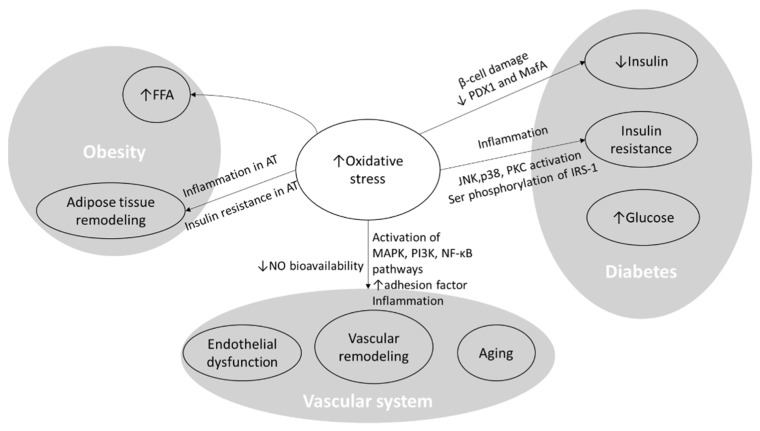
The effects of oxidative stress on obesity, diabetes, and the vascular system. FFA: free fatty acid; AT: adipose tissue; PKC: protein kinase C; NF-κB: nuclear factor-κB; NO: nitrite oxide; MAPK: nitrogen-activated protein kinase, PI3K: phosphoinositide 3-kinases; JNK: Jun *N*-terminal kinase; IRS-1: Insulin receptor substrate 1; ↑: increase; ↓: decrease.

Genome, epigenome, and transcriptome; the structure and function of molecules, organelles, and cells; molecular and cellular interactions; and signaling pathways are all different levels affected by high levels of oxidative stress. Oxidation and the consequent damage to DNA, proteins, and lipids, as well as mitochondrial dysfunction, are some examples of these effects [43]. All components of MetS pathophysiology, as well as the vascular system, are affected by high levels of oxidative stress. These effects are briefly demonstrated in Figure 3.

As one of the main alterations induced by oxidative stress, lipid peroxidation leads to the production of MDA, propanal, hexanal, and 4-hydroxynonenal (4-HNE), which can induce molecular damage. MDA and 4-HNE are mutagenic and toxic products of lipid peroxidation, respectively [44]. In particular, PUFA autoxidation, or the isoprostane pathway of lipid peroxidation (IPLP), is one of the main pathways that can produce acutely toxic molecules, such as γ-ketoaldehydes [39]. Because of their contribution to the oxidant–antioxidant balance, antioxidants can be considered significant molecules affected by ROS. Under hyperglycemic conditions, studies show a decrease in the activity of antioxidant enzymes, including CAT and GPx, by ROS, and the inactivation of copper (Cu)/zinc (Zn)-SOD by the glycation of specific lysine residues [8]. The other important molecule affected by high levels of oxidative stress is mitochondrial manganese superoxide dismutase (Mn-SOD); this molecule is important because of its antioxidant role. The interaction of oxidants with Mn-SOD results in the inactivation of Mn-SOD and the inhibition of its toxification [45]. In addition, matrix metalloproteinases are the other group of molecules affecting high ROS levels. The overactivity of these molecules at high levels of ROS can cause tissue damage due to the polymerization of hyaluronan and degradation of proteoglycans and collagen [43].

Due to its significant role in vascular function, NO appears to be one of the most important molecules affected by oxidative stress un the pathogenesis of vascular diseases. The balance between NO and oxidative stress facilitates appropriate vascular function. The interaction of NO and oxygen-free radicals leads to a decrease in NO bioavailability and peroxynitrite formation. In the next step, the interaction between peroxynitrite and NO synthase leads to the oxidation of tetrahydrobiopterin (BH4) and the formation of NO synthase uncoupling [7,46]. There are different mechanisms that lead to reduced eNOS activity and result in a decrease in NO bioavailability; some of these are the modification (OGlcNAcylation) of eNOS, activation of PKC, impairment of signaling pathways related to soluble guanylate cyclase due to hypercholesterolemia, and the interaction of AGEs and their soluble receptor [35].

In MetS, there are three main groups of cells and tissues affected by elevated oxidative stress: (i) muscle, adipose tissue, and liver; (ii) pancreatic β-cells; and (iii) endothelial cells. Since the first group is related to insulin-dependent metabolic activities, the main change in this group is insulin resistance [16]. The direct mechanism of inducing insulin resistance in skeletal muscle cells is the serine phosphorylation of insulin receptor substrate 1 (IRS-1) by the stress-response kinases (JNK, p38, and PKC) activated by ROS [38]. Furthermore, pro-inflammatory adipose tissue can increase oxidative stress and lipid peroxidation, leading to insulin resistance [8]. TNF-α, leptin, FFAs, and resistin are the components of adipose tissue remodeling in obesity that can contribute to oxidative stress-induced insulin resistance [16]. In addition, the accumulation of lipid peroxidation products in cells, such as 4HNE and oxysterols, can activate NF-κB signaling and result in inflammation and insulin resistance [47]. Leptin, visfatin, and FFAs are other activators of NF-κB signaling [8,16]. In adipocytes, mitochondrial dysfunction-induced oxidative stress can also lead to disturbed insulin signaling in the cell and, consequently, insulin resistance [8]. Diminished antioxidant defenses in cells is another mediating factor inducing insulin resistance in cells [36,38]. The down-regulation of Mn-SOD in skeletal muscle cells in patients with obesity is one example. In addition to enzyme antioxidants, the down-regulation of transcription factors associated with antioxidant proteins, such as nuclear factor erythroid 2-related factor 2 (Nrf2), is one of the important factors in inducing insulin resistance due to oxidative stress [38].

Pancreatic β-cells constitute the second most important site impacted by elevated oxidative stress. The importance of these cells is due to their role in insulin production. Furthermore, the increased sensitivity of these cells to oxidative stress is related to their lower levels of antioxidants such as SOD, GPx, CAT, and thioredoxin (TR) [16,36,47]. Mitochondrial dysfunction, endoplasmic reticulum (ER) stress, and DNA damage are significant consequences of oxidative stress in β-cells. The activation of AMP-activated protein kinase (AMPK) and JNK and down-regulation of the mammalian target of rapamycin (mTOR), as a result of increased oxidative stress, can lead to decreases in the mass, proliferation, apoptosis, and dedifferentiation of β-cells [48]. The damaging and dysfunction of these cells indicate excessive ROS generation, a reduction in ATP production, and insulin secretion [47]. It should be noted that appropriate levels of H_2_O_2_ up-regulate insulin secretion; however, the excessive generation of this molecule, and the ability of β-cells to detoxify them, result in inhibited insulin secretion [38]. The decreased action on ATP-sensitive K^+^ channels due to decreased ATP production is another mechanism involved in β-cells’ impairment of insulin secretion [40]. Furthermore, elevated ROS levels can also negatively regulate insulin gene expression through redox-sensitive transcription factors such as pancreatic and duodenal homeobox 1 (PDX1) and homolog A of the V-Maf avian musculoaponeurotic fibrosarcoma oncogene (MafA) [40].

The third site affected by high levels of oxidative stress is the vascular system, and endothelial cells in particular. The activation of signaling pathways such as MAPK, phosphoinositide 3-kinases (PI3K), and NF-κB induces inflammation, the invasion of monocytes into the vessel wall, the up-regulation of adhesion factors, and the proliferation and triggering of aging mechanisms. Particularly at the cellular level, the proliferation, hypertrophy, and apoptosis of vascular smooth muscle cells (VSMCs) induced by high oxidative stress manifest as vascular remodeling [43,49].

Accelerated aging in response to high levels of oxidative stress is one of the significant points in the development of vascular diseases. There are different mechanisms involved in the aging of endothelial cells, triggered by high levels of oxidative stress, some of which are molecular damage, mitochondrial dysfunction, inflammation, and telomere shortening [45]. In particular, age-related telomere dysfunction in endothelial cells leads to endothelial cell senescence. This can cause high levels of oxidative stress and inflammation and contribute to arterial and metabolic dysfunction [50]. The aging of endothelial cells results in some structural changes in the vasculature, including luminal enlargement, the thickening of the intima media, and an increase in both endothelium permeability and vascular stiffness. Furthermore, there are some functional changes, such as diminished vasodilation, high sensitivity to vasoconstrictors, and impaired angiogenesis and repair mechanisms [51].

It seems that elevated levels of oxidative stress and the subsequent effects are key points in the pathophysiology of MetS and the development of CVD. Therefore, targeting them can be a promising tool for the controlling and treatment of MetS.

## 5. Antioxidant Therapy for MetS Patients and Their Associated CVDs

Since there is an imbalance between oxidants and antioxidants in patients with MetS, antioxidants can be effective tools in helping to adjust this balance and protect these patients from complications such as cardiovascular diseases. However, selecting the best option for therapy is challenging, because, in this complex condition, there are several different mechanisms related to obesity, adipose tissue remodeling, dyslipidemia, diabetes, insulin resistance, hyperinsulinemia, hypertension, and endothelial dysfunction. Therefore, the best therapeutic tools can be ascertained by considering the different aspects of this complex network. In order to achieve this aim, this section is designed to review not only antioxidant functions, but also the role of antioxidants in obesity, dyslipidemia and adipose tissue remodeling, diabetes and insulin metabolism, hypertension, and endothelial function.

Polyphenols, such as resveratrol; flavonoids, such as quercetin or anthocyanin; carotenoids; N-acetylcysteine; melatonin; L-arginine; vitamins, such as vitamins C and E; and minerals, such as zinc, cooper, and selenium are the main antioxidant supplements considered for MetS and its complications (Table 2).

### 5.1. Polyphenols

Polyphenols, an important group of antioxidants, are divided into two classes: non-flavonoids and flavonoids. Resveratrol is an example of a non-flavonoid antioxidant that is abundant in sources such as grapes, apples, blueberries, plums, wine, and peanuts. the main antioxidant and anti-inflammatory functions of resveratrol are reducing H_2_O_2_ production, preventing ROS generation after the suppression of the MAPK signaling pathway, and modulating inflammation by down-regulating the protein kinase A (PKA) and Akt/PKB pathways [6,98,99]. Furthermore, white adipose tissue (WAT) can act as an anti-obesity agent in BAT remodeling, affecting lipogenesis and lipolysis and improving mitochondrial function in adipose tissue [6,100,101]. Resveratrol can also improve both β-cell insulin secretion and hyperglycemia [53,94]. The anti-atherogenic functions of resveratrol are the inhibition of ET-1 synthesis, stimuli-induced smooth muscle cell proliferation, and arterial stiffness. Furthermore, the overexpression of eNOS and stimulation of its activity by resveratrol lead to enhanced NO production in endothelial cells [6,98]. Resveratrol also has an anti-hypertensive effect by moderating blood pressure through sodium excretion via the kidneys [53].

### 5.2. Flavonoids

Flavonoids are a group of antioxidants that include flavonols, flavones, flavanones, flavanols, anthocyanins, and isoflavones. Foods such as citrus fruits, blueberries, blackberries, onions, peppers, soybeans, green beans, kudzu root and alfalfa sprouts, a variety of teas, cocoa, dark chocolate, oregano, and parsley are natural sources of these antioxidants. In addition to their antioxidant functions, such as ROS scavenging and endogenous antioxidant defense improvement, flavonoids affect obesity and lipid metabolism, insulin and glucose metabolism, and hypertension, as well as the cardiovascular system. The related effects of obesity and dyslipidemia include decreased body weight, BMI, total cholesterol, LDL, oxidized LDL (ox-LDL), and visfatin, as well as elevated adiponectin [53,82], although some studies show that body weight and BMI are not altered by consuming flavonoids [82]. Flavonoids’ effects on insulin are increased insulin sensitivity and signaling, and reduced serum insulin levels and insulin intolerance. Flavonoids can also attenuate blood pressure and improve endothelial function and NO production [53,82].

The most abundant dietary flavonol is quercetin. As a potent ROS scavenger, quercetin prevents oxidative stress-induced macromolecular damage, such as lipid peroxidation. Furthermore, the inhibition of NOX2 by quercetin prevents ROS production. One of the other antioxidant functions of quercetin is chelating Cu^2+^ and Fe^2+^. Quercetin also improves the endogenous antioxidant capacity by enhancing the expression of SOD, CAT, and GSH. The anti-inflammatory mechanisms of quercetin include the suppression of NLRP3 (nucleotide-binding domain, leucine-rich family, pyrin domain-containing-3), inflammasomes, neutrophil infiltration, and the NF-κB and ROS/AMPK pathways, as well as the modulation of inflammatory mediators and TNF-α-activity [6,102]. Thermogenesis, the browning of WAT, and the modulation of inflammation in BAT are the anti-obesity features of quercetin [103,104]. Quercetin can also modulate hyperglycemia and insulin levels, improving insulin resistance. The anti-hypertensive, anti-atherosclerotic, and vasodilatory effects of quercetin, as well as its senolytic function in selectively removing aging endothelial cells, means that this antioxidant is able to protect the cardiovascular system [6,81]. In diabetic patients, anthocyanin supplements can improve glucose and lipid metabolism, as well as inflammation [105]. By improving dyslipidemia and reducing body weight and BMI, this antioxidant can be beneficial in attenuating the obesity-related mechanisms involved in MetS pathogenesis [53,82].

### 5.3. Carotenoids

Carotenoids are another group of antioxidants that are pigmented compounds synthesized by plants and microorganisms that animals cannot produce. Fruits and vegetables are the primary sources of carotenoids in the human diet. Some examples of food sources rich in carotenoids are tomatoes, carrots, spinach, broccoli, green beans, tangerines, and papayas [53]. It seems that there is an inverse association between MetS and total carotenoids, indicating the importance of this group of antioxidants in the condition [92]. The two carotenoids that are most commonly found in food, β-carotene and lycopene, are stored in adipose tissue and can affect various physiological adipocyte functions. Studies also indicate that there is a positive correlation between β-carotene and adiponectin. Furthermore, carotenoids’ reducing of visceral fat, LDL, and TG can moderate dyslipidemia and obesity in MetS [53]. The down-regulation of the NF-κB and MAPK signaling pathways by carotenoids leads to inflammation modulation and activates reverse cholesterol transport, resulting in suppressed foam cell formation [96]. Carotenoids can also reduce glucose and insulin resistance, which is important in MetS management [53,91].

### 5.4. N-acetylcysteine

N-acetylcysteine (NAC), the acetylated form of the amino acid L-cysteine, is an antioxidant that is a precursor of glutathione, of which the allium plant is a natural source [91]. The antioxidant activity of NAC depends on increased endogenous concentrations of total glutathione, the elimination of free radicals, and suppressed ROS generation [95]. In addition to its antioxidant activity, it has an anti-inflammatory and vasodilatory role. NAC can also improve dyslipidemia by increasing HDL and decreasing TG. By stimulating insulin production, NAC has been shown to reduce blood sugar levels [97,106].

### 5.5. Melatonin

Due to the role of melatonin in both circadian rhythm regulation and lipid metabolism, it has been reported to be an appropriate supplement for many diseases such as diabetes, CVDs, and MetS [74]. Natural sources of melanin are eggs, fish, and nuts, as well as some cereals, germinated legumes, seeds, and mushrooms [75]. The conversion of WAT to BAT and the increase in BAT thermogenesis in obese rats make this antioxidant a suitable tool for controlling obesity [6]. Since insulin secretion is directly affected by melatonin, the circadian rhythms of insulin and melatonin are opposites [75]. Therefore, melatonin supplementation leads to an improvement in insulin resistance due to circadian rhythm regulation. Furthermore, there is some evidence that melatonin may be beneficial in modulating HbA1C and hyperglycemia [77]. Melatonin also down-regulates some inflammatory cytokines, like TNF-α, interferon-γ (IFN-γ), IL-2, and IL-6; inflammatory pathways, such as NF-κB; and pro-inflammatory genes, such as inducible NOS (iNOS), TNF-α, and cyclooxygenases (COX-1 and COX-2) [74,76]. Furthermore, melatonin can be beneficial for MetS, due to its role in lowering blood pressure [74,77]. The antioxidant, anti-inflammatory, antithrombotic, and anti-atherogenic activities of melatonin make it a potential therapeutic tool for CVDs [75,76].

### 5.6. L-arginine

Studies show that L-arginine (L-Arg) has a regulatory effect on the metabolism of carbohydrates and lipids. Since L-Arg deficiency contributes to NOS uncoupling and ROS generation, L-Arg appears to be an important supplement in improving NO production and endothelial function and preventing ROS generation [79,107]. L-Arg can also attenuate cholesterol, LDL, and TG levels and increase levels of adiponectin [81,82]. Furthermore, the decrease in macrophage infiltration and the production of their pro-inflammatory cytokines, in addition to the suppression of the NF-κB pathway, constitute the anti-inflammatory functions of L-Arg [78,107]. L-Arg has been reported to participate in glucose metabolism and insulin sensitivity [80].

### 5.7. Vitamins C and E

Fruits like kiwis, oranges, and grapefruit, and fruiting vegetables, such as tomatoes, are the main sources of vitamins C and E; other sources include vegetable oils, non-citrus fruits, olives, and nuts. These vitamins can control both oxidative stress and inflammation by neutralizing free radicals and reducing levels of IL-6 and hs-CRP, leading to a decrease in inflammation-induced oxidative stress. In addition to moderating inflammation, oxidative stress, and lipolysis, vitamin C reduces markers of hypoxia and endoplasmic reticulum (ER) stress and stimulates adiponectin secretion and XO activity [6,58,108]. The increase in insulin production and GLUT-4 levels by vitamin C can help modulate glucose metabolism in patients with MetS. Vitamin C improves endothelial function and reduces systolic blood pressure [61,109]. The anti-aging role of this vitamin includes cell protection from oxidative stress, chromatin damage, and telomere shortening [110].

Based on an animal study in mice, vitamin E supplementation can decrease oxidative stress, as well as IL-6, TNF-α, leptin, resistin, PAI-1, and collagen deposition, in visceral adipose tissue [6]. In addition to inhibiting lipid peroxidation and reducing total cholesterol, vitamin E also attenuates hypertension, platelet aggregation, and levels of adhesion molecules such as intercellular adhesion molecule-1 (ICAM-1), vascular cell adhesion protein 1 (VCAM-1), and E-selectin, improving NO bioavailability [52,53]. As a result, it seems vitamin E intake can help in preventing CVD [111].

### 5.8. Minerals

In terms of minerals, it seems that zinc, cooper, and selenium can be appropriate antioxidant supplements for MetS. Zinc is known for its crucial role in the appropriate functioning of cells. Zinc deficiency can affect many parts of the body, including the epidermal, gastrointestinal, central nervous, immune, skeletal, and reproductive systems [61]. Food sources rich in zinc are meat and sea food (oysters). As a cofactor for SOD enzymes, zinc acts as a ROS scavenger by detoxifying O^2^ radicals into H_2_O_2_, protecting cell membranes, and inhibiting lipid peroxidation [112]. Furthermore, zinc enhances the expression of peroxisome proliferator-activated receptor a (PPAR-α), which is a crucial factor for regulating inflammation and lipoprotein and glucose metabolism. As an anti-inflammatory molecule, zinc plays a negative modulating role in the NF-κB pathway. PPAR-α restricts NF-κB activation [65]. Furthermore, zinc can up-regulate the insulin signaling pathway in T2D and can also stimulate glucose uptake by inducing GLUT1 and GLUT4 expression [63]. According to some studies, zinc deficiency is associated with an increased risk of CVD and hypertension. Due to its cofactor role for the CuZn-SOD enzyme, the association between zinc and hypertension hinges on maintaining adequate levels of NO. It should be noted that zinc is toxic when consumed excessively and can cause an undesirable HbA1c elevation and high blood pressure. Prescribing zinc for patients with MetS is thus challenging [112].

Copper is typically obtained from various foods, including milk, meat, seafood, vegetables, and fruits [68]. Copper ions are involved in radical reactions, catalyzing superoxide to hydrogen peroxide; hydroxyl radicals, as well as oxidative modifications of LDL, are some examples of this [10]. Copper plays an important role in the modulation of many enzymes such as ZnCu-SOD, lysyl oxidase, and cytochrome c oxidase; copper deficiency is thus associated with many disorders [113]. One of the common problems associated with the use of zinc supplementation is copper deficiency, so it is better to use fewer copper doses with zinc supplementation [112]. Copper supplementation is also challenging, due to the possible risk of inducing undesirable effects such as inflammation, oxidative stress, and increased cardiovascular risk [69].

The antioxidant functions of selenium pertain to increased antioxidant activity and glutathione levels and the suppression of lipid peroxidation. Inflammation modulation, due to the down-regulation of TNF-α, IL-1β, and PGE2, turns selenium supplementation into an anti-inflammatory tool. Attenuating dyslipidemia and resistance to leptin, as well as improving hyperglycemia and insulin resistance, are the other effects of this mineral antioxidant [53,71,73].

## 6. Epigenetic Role in MetS: A Bridge between Environment and MetS Pathophysiology

Since environmental factors such as diet or physical activity are considered significant etiological factors for MetS, it seems that epigenetic mechanisms play an important role in the condition’s pathophysiology [114]. Epigenetic mechanisms, such as DNA methylation, histone modification, chromatin remodeling, and noncoding RNA (ncRNA), are involved in the pathophysiology of MetS. Liver, skeletal muscles, pancreatic islets, adipose tissue, and blood are the main sites of epigenetic changes during MetS. For example, DNA methylation is related to developing obesity and changes in weight. In diabetes, DNA methylation is associated with diabetic complications [115]. In particular, the methylation site cg19693031 in the TXNIP gene is inversely associated with fasting glucose levels; furthermore, cg06500161 in the ABCG1 gene, which is involved in lipid transport, is associated with serum triglycerides and waist circumference [116]. Decreased CPT1A methylation is associated with MetS through the regulating role of CPT1A in mitochondrial fatty acid oxidation. The DNA methylation of LINE-1 is also associated with MetS via the induction of metabolic changes in visceral adipose tissue [117]. There appears to be an interaction between DNA methylation and oxidative stress. Furthermore, in the development of atherosclerosis, DNA methylation can regulate the chronic inflammatory response in the arteries [118].

Studies demonstrate that histone deacetylases (HDACs) are associated with different components of MetS. A high-fat diet can up-regulate the hypothalamic expression of HDACs, which consequently induce obesity, affecting leptin sensitivity and resistance. Therefore, the inhibition of some HDACs, such as HDAC6, can act as a leptin sensitizer and anti-obesity factor and can thus be considered a potent therapeutic tool [114,119]. Both HDAC3 activity and its mRNA level are correlated with insulin resistance and inflammation in diabetic patients. HDAC9 is also related to glucose metabolism. Sirtuins (SIRTs), which are a class of HDACs, are related to glucose metabolism and insulin resistance. In particular, the absence of deacetylation by SIRT1, SIRT2, and SIRT6 can contribute to obesity and diabetes [117]. Furthermore, the inhibition of the HDAC–Ang II–vascular contraction axis leads to reduced blood pressure by inhibiting angiotensin II production [120]. Since histone deacetylase inhibitors (HDACi) can regulate gene expression via epigenetic changes, their antioxidant, anti-inflammatory, and antithrombotic functions turn them into therapeutic options for metabolic and cardiovascular diseases [121]. Inhibitors of zinc-dependent HDACs (class I HDAC (HDAC1, 2, 3, 8), class IIa HDAC (HDAC4, 5, 7, 9), class IIb HDAC (HDAC6, 10), and class IV HDAC (HDAC11)) are beneficial for controlling hypertension [122]. Valproic acid (VPA), sodium phenylbutyrate (PBA), and trichostatin A (TSA) are some HDACis that can affect both diabetes and obesity by improving insulin resistance, inflammation, and gluconeogenesis, as well as decreasing fat accumulation [117].

Chromatin remodeling is another significant change during the pathophysiology of MetS, with the interaction of the β-cell regulator PDX1 with NuRD and SWI/SNF complexes in β-cells being just one example. Through the significant presence of the SWI–SNF complex, Pdx1 interaction with the Ins gene enhancer results in mature β-cell functionality and the proliferation of pancreatic progenitor cells. On the one hand, blood glucose in diabetic patients affects the formation of the SWI/SNF chromatin remodeling complex; on the other hand, this complex changes the expression of both the transcription factor GATA4 and endothelin, thus affecting cardiac function [114,115].

MiRNA-based epigenetic changes are associated with the inflammation in adipose tissue induced by obesity, insulin resistance, and T2D [119]. miR-17-5p and miR-519d are related to obesity and lipogenesis, miR-17-5p is negatively associated with BMI and waist circumference, and it also seems that miR-17-5p is associated with central adiposity [3]. miR-377, which acts as a significant regulator of adipogenesis, negatively regulates SIRT1 by targeting its mRNA, resulting in the development of both inflammation caused by obesity and insulin resistance. As a consequence of epigenetic changes in miRNA, the suppression of SIRT1 also contributes to reduced insulin sensitivity. Through its effect on adipocyte differentiation, metabolic homeostasis, and insulin signaling, miR-221 is one of the examples of miRNAs involved in this mechanism. miR-22-3p also affects the inhibition of adipogenic differentiation by suppressing HDAC6 [117]. Furthermore, miR-375 regulates insulin secretion from the β-cells and the let-7 family participates in insulin sensitivity and glucose metabolism [3].

Some of the antioxidants considered as therapeutic tools for MetS also participate in epigenetic changes. For example, Nrf2, which is one of the antioxidant functions of vitamin C, functions by demethylating DNA mediated by ten-eleven translocation (TET) proteins. Furthermore, vitamin E reportedly down-regulates DNA methyltransferase-1 (DNMT1) expression and consequently up-regulates PPARγ [118]. Quercetin performs its epigenetic regulation by suppressing three epigenetic mechanisms, including DNA methylation (via the inhibition of DNMT1), HAT activity, and HDACs [123].

According to some studies, the regulation of HATs, HDACs, and SIRT1 activity can act as an epigenetic regulator of inflammation by influencing NF-κB signaling. Many phenolic compounds can act as anti-inflammatory agents due to this epigenetic modification [124]. Furthermore, dietary flavonoids have anti-diabetic effects and appear to be efficient in preventing diabetes [125]. Resveratrol can also affect the activation of SIRT1, making this antioxidant efficient in improving metabolic disorders. Other epigenetic elements affected by resveratrol include DNMT and lysine-specific demethylase-1 (LSD1) [126]. Taken together, it appears that antioxidants are beneficial not only because of their direct effects on oxidative stress, inflammation, lipid and glucose metabolism, obesity, and diabetes, but also because they can affect MetS components through epigenetic changes.

## 7. The Pathophysiological Network of Metabolic Syndrome and the Challenges in Its Management

Since MetS is a multidimensional condition that contains different components, understanding the interaction between these is the first step in understanding its role in CVD development. There is an interaction between elevated FFA levels caused by obesity and decreases in glucose uptake and insulin secretion from β-cells, resulting in hyperglycemia and insulin resistance. This interaction seems mutual, because insulin resistance also affects dyslipidemia and lipolysis, leading to increased FFA. The other interaction is between obesity and hypertension, represented by physical pressure on adipose tissue in the kidneys, increased vascular tonicity, and the activation of the sympathetic system and RAAS. Insulin’s interaction with the vascular, renal, and nervous systems also leads to hypertension, thus demonstrating the interaction between insulin and hypertension in MetS.

The second step is to illustrate how this MetS pathophysiological network contributes to the development of CVDs. The interactions between the components of MetS and the vascular system can demonstrate this. Pathophysiological changes during obesity induce reduced NO, vascular remodeling, endothelial aging, vascular dysfunction, and thrombosis. At the same time, insulin resistance and hyperglycemia, as well as hypertension, promote vasoconstriction, endothelial dysfunction, and a prothrombotic state.

The main mechanisms involved in the pathophysiology of the MetS network and its interaction with the vasculature seem to be inflammation and oxidative stress. There is a mutual interaction between these key mechanisms and the components of MetS. On the one hand, overnutrition, obesity-induced adipose tissue remodeling, insulin level, and hypertension lead to elevated oxidative stress. On the other hand, elevated oxidative stress induces insulin resistance in muscles, adipose tissue, and the liver, as well as β-cell damage and reduced insulin production. Furthermore, it seems that a high level of oxidative stress is one of the main factors involved in endothelial dysfunction. Therefore, treating MetS patients with antioxidants may be a therapeutic approach to preventing CVD development.

In prescribing antioxidants for a complex condition such as MetS, it is not only their antioxidant function that matters, but their other activities, such as their effects on glucose levels, insulin, dyslipidemia, adipose tissue remodeling, and hypertension, should also be considered. Moreover, there are some challenges in antioxidant therapy. The first challenge is in considering the pro-oxidant–antioxidant balance, rather than oxidative status alone. Since the excessive use of antioxidants can lead to an effect called “the oxidative effect of antioxidants”, it is important to maintain this balance in order to prescribe the appropriate dose of antioxidants [6]. The second challenge pertains to holistic management and personalized medicine. Since MetS has a multidimensional pathophysiology, for successful treatment, a holistic approach considering all of these different dimensions is crucial. Since the pathophysiology of MetS is a complex network, ignoring a single part of it during treatment would allow for this syndrome to redevelop. Therefore, a holistic and comprehensive treatment strategy is needed to simultaneously cover all components. Based on this review, the recommended strategy includes two parts: the first targets oxidative stress and inflammation as key points of this network, while the second targets each pathophysiological component, including obesity, diabetes, and hypertension. As mentioned previously, in MetS, some antioxidants have additional effects besides their antioxidant and anti-inflammatory functions. Therefore, it seems that prescribing antioxidants can satisfy both parts of this strategy. Additionally, the different weight of each component for individual patients indicates the need for personalized medicine on a case-by-case basis. For example, in patients with PAD, since adequate coronary and peripheral artery perfusion depend on blood pressure, the treatment of hypertension in patients with MetS needs special consideration [33,34]. Furthermore, the treatment of PAD as a multifactorial condition requires therapies based on the patients’ underlying disease. Therefore, therapies such as anticoagulants, antiplatelet agents, anti-hypertensives, anti-diabetic agents, and statins, in addition to the appropriate amount of antioxidants, can be useful [125]. Therefore, a comprehensive and personalized approach, in which all components of MetS are considered based on their impact, and in which the oxidant–antioxidant balance is considered as the central point, seems to be an appropriate therapeutic approach for MetS and its associated CVDs.

## 8. Conclusions

In general, due to the nature of MetS as a multidimensional condition, it seems that the best management approach is one that is simultaneously holistic and personalized, covering all components of MetS including obesity, adipose tissue remodeling and dyslipidemia, diabetes and insulin resistance, and hypertension. Since CVD is one of the main complications induced by MetS, improving endothelial function should also be considered as one of the main therapeutic targets. On the one hand, through different mechanisms, all components of MetS are involved in elevating oxidative stress. On the other hand, this elevated oxidative stress can exacerbate obesity, dyslipidemia, diabetes, insulin resistance, and hypertension, as well as affect endothelial function. Therefore, the important role of oxidative stress in the pathophysiology of MetS and development of CVDs indicates that managing its levels and inflammation should play a central role in therapeutic strategies. This paper attempted to shed necessary light on the exact roles of various antioxidants, in the different components of MetS and endothelial cells, in order to suggest an appropriate antioxidant therapy.

## Figures and Tables

**Table 1:** The level of evidence of the studies included in this review.

Case Report	Cross Sectional Study	RCT ^1^	Meta-Analysis and Systematic Review
13	7	6	8

^1^ Randomized control trial.

**Table 2:** The role of antioxidants in MetS and endothelial dysfunction.

Antioxidants	Sources	Functions					
		Antioxidant Function	Anti-Inflammatory Role	Obesity, Dyslipidemia, Adipose Tissue Remodeling	Hyperglycemia, Insulin Resistance, Hyperinsulinemia	Hypertension	Endothelial Dysfunction
**Vitamin E**	Vegetable oils, safflower seed oil, soy oil, palm oil [52]	Scavenging free radicals [53] ↑ NO bioavailability ↓ NOX activity [54] Inhibition of lipid peroxidation [54]	↓ CRP level ↓ Pro-inflammatory cytokines (IL-1 and 6, TNF) ↓ PGE2 synthesis ↓ Chemokine IL-8, PAI-1 levels [52]	Inhibition of cholesterol synthesis ↓ LDL oxidation [55]	Improving insulin sensitivity ↓ Plasma glucose [56] ↓ HbA1c [57]	↓ Blood pressure	↓ Monocyte adhesion to endothelium ↓ E-selectin, ICAM-1, and VCAM-1 ↓ Platelet aggregation [52] ↓ Risk of CVD morbidity and mortality [54] protecting endothelial cells from chromatin damage protecting endothelial cells from telomere shortening [6]
**Vitamin C**	Cherries, wild rose, blackcurrant, guava, peppers, brussels, broccoli, grapefruit, pomelo, lemon, orange, lime [58]	↓ ROS production ↑ NO bioavailability [54] ↓ XO activity ↓ NOX activity ↑ SOD activity ↑ GPx activity ↑ GR activity ↑ TrxR activity Preventing lipid peroxidation [58]	↓ IL-2,6,12, IFN-γ Activation of Nrf2 Suppressing NF-κB, TNF-α pathway ↓ CRP [58]	↑ HDL ↓ Cholesterol, LDL and TG [59]	↓ Plasma insulin ↓ Plasma TGL ↓ HbA1c ↓ FBS [60]	↓ Systolic blood pressure [61]	↓ Endothelial dysfunction [62] ↓ Risk of CVD morbidity and mortality [54]
**Zinc**	Milk, cheese, red meat, or liver [61]	↑ CuZn-SOD activity [63] ↑ eNOS expression levels ↑ NO availability [64]	Suppression of the NF-B pathway ↑ PPAR-α expression [65]	↑ Leptin synthesis Improve leptin sensitivity [66]	↓ Glucagon secretion ↓ Insulin resistance ↑ Insulin sensitivity ↑ GLUT4 Translocation [63]	↓ Systolic blood pressure [67]	Prevention of endothelial dysfunction [64]
**Copper**	Milk, meat, seafood, vegetables, fruits [68]	Scavenging free radicals ↑ CuZn-SOD activity [69]	Activation of PPAR-α signaling [68]	↓ Total cholesterol ↓ LDL [69]	↓ Plasma glucose [69] ↓ Intramuscular fat accretion [68]	↓ Diastolic blood pressure [69]	Benefited the fibers of the blood vessel wall by maintaining its toughness and suppleness [70]
**Selenium**	Fish, such as tuna and mackerel, animal foods, cereals, plant sources, such as garlic, onions, and broccoli [71]	↑ NO bioavailability ↓ Lipid peroxidation ↑ Antioxidant capacity in liver and kidney [72] ↑ GSH in the RBCs and liver ↑ Antioxidant enzyme activities (↑ GPx activity) [73] ↓ MDA [74]	↓ TNF-α, IL-1β, PGE2 [68] ↓ Leukocyte and neutrophil count in circulation ↓ CRP, IL-6 ↓ MDA Inhibition of NF-B [74]	↓ Leptin resistance Improving hyperlipidemia [73] ↑ HDL ↓ LDL ↓ TG ↓ Ratio of total cholesterol to HDL cholesterol [71]	↓ Blood glucose ↓ Diabetes prevalence [71] ↑ Insulin secretion ↓ Insulin resistance [73]	High levels of selenium: ↑ incidence of hypertension	Inhibition of platelet aggregation [72]
**Melatonin**	Eggs and fish, nuts, some cereals, germinated legumes or seeds, mushrooms [75]	Scavenging of free radicals ↓ Lipid peroxidation ↑ NO synthesis ↓ MDA Activation of SOD, CAT, and GR [74] Activation of SIRT1/Nrf2 signaling pathway ↓ Oxidative stress damage [75]	Down-regulation of chemokine expression Inhibition of the NF-κB phosphorylation of PI3K/Akt, p38, ERK, JNK and MAPK [75] ↓ NOX ↓ Pro-inflammatory mediators (COX-2, TNF-α, iNOS) [76]	↓ LDL ↑ HDL [74] ↓ Leptin resistance [77] ↓ Total cholesterol, TG, and ox-LDL ↓ Body weight ↓ Intra-abdominal visceral fat deposition [76]	Improvement in insulin resistance ↓ Hyperinsulinemia ↓ Hyperglycemia ↓ HbA1c ↓ Incidence of T2D [77]	↓ Blood pressure [74]	Prevention of endothelial dysfunction [76] ↓ E-selectin, ICAM-1, and VCAM-1 [76] Inhibition of platelet aggregation Alteration of levels/activity of proteins involved in the coagulation cascade [77]
**L-arginine**	Seafood, watermelon juice, nuts, seeds, algae, meats, rice protein concentrate, and soy protein isolate [78]	↑ ↓ ROS production ↑ SOD activity ↓ Lipid peroxidation [79] NO bioavailability ↓ MDA [80]	↓ TNF-α, IL-1β, and IL-6 secretion [78]	↓ Total cholesterol, LDL and TG ↑ HDL ↑ Adiponectin ↓ Body fat ↓ FFA ↓ Leptin [80]	↓ Risk of diabetes ↑ Insulin secretion ↓ Blood glucose ↑ Insulin sensitivity [80]	↓ Blood pressure [80]	Improvement in endothelial function [80]
**Quercetin (Flavonol)**	Citrus fruits, apples, grapes, dark cherries and dark berries, onions, parsley and sage, tea, olive oil, red wine [53], mangoes, buckwheat, plums, and tomatoes [6]	ROS scavenger Inhibition of lipid peroxidation Inhibition of ROS production up-regulating the expression of SOD, CAT, and GSH ↑ TAC and GPX ↓ MDA Prevention of ROS production by inhibition of NOX2 Cu^2+^ and Fe^2+^ chelation [6]	Inhibition of neutrophil infiltration Inhibition of NLRP3 pathways Inhibition of NF-κB pathways Inhibition of ROS/AMPK pathways ↓ IL-6 and TNFα [6]	↓ Inflammation in BAT Promoting thermogenesis and browning of WAT [6] ↓ Ox-LDL ↓ Body weight [53] ↓ TG ↓ Cholesterol ↑ Adiponectin ↓ Leptin ↑ HDL [81] ↓ Waist circumference [82]	↓ Blood glucose ↓ Insulin resistance ↑ Insulin ↓ HbA1c [81] ↑ β-cell number [82]	↓ Systolic blood pressure [53]	Removal of endothelial cells aging Vasodilatory effect due to up-regulation of eNOS Anti-atherosclerosis effects Reduction in CVD [6]
**Flavones**	Dried oregano, dried parsley [53]		↑ Activation of PPARs ↓ MCP-1 ↓ TNF-α ↓ INF-γ ↓ IL-1β ↓ IL-6 [83]	↑ Adiponectin ↓ Body weight ↓ TG ↓ Cholesterol ↓ LDL ↓ V-LDL ↓ Apo-B [83]	↑ Glucose tolerance glycemic control improvement ↓ Insulin resistance Regulation of GLUT4 expression [83]		Blockage of macrophage foam cell formation [83]
**Flavanones**	Grapefruit juice and cooked tomato [53]	Stimulation of the antioxidant defense system and apoptosis [84] ↑ Antioxidant Capacity [82]	↓ CRP ↓ TNF-α ↓ IL-6 [82]	↓ LDL ↑ HDL ↓ TG ↓ Cholesterol ↓ ApoB ↑ Adiponectin [82]	↑ Insulin sensitivity ↑ Insulin signaling [53] ↓ Blood glucose ↓ Insulin resistance ↓ C-peptide ↓ Glucose intolerance ↓ HbA1c ↑ GLUT4 [82]	↓ Blood pressure [82]	↑ Endothelium-dependent vasodilation ↑ NO production ↓ IL-6 ↓ E-selectin ↓ P-selectin ↓ VCAM-1 ↓ ICAM-1 [82]
**Catechins (Flavanol)**	Brewed green tea, black tea, blueberries, fava beans, cocoa, and dark chocolate [53]	↓ ROS formation and NOX activity ↑Phosphorylation of eNOS and ↑ NOS production using the PI3k-dependent pathway ↑ GSH [53] ↑ NO and consequently decrease in formation of reactive oxygen and nitrogen species [24]	Inhibition TNF-α- mediated NF-κB, and MAPKs activation [85]	↓ Body weight ↓ BMI ↓ Cholesterol ↓ LDL ↓ TG [53]	↓ Blood glucose ↓ Glucose intolerance ↑ Insulin sensitivity [53]	↓ Blood pressure [53]	↓ Endothelial dysfunction [53]
**Anthocyanins**	Blueberries, strawberries, pomegranates, wine, asparagus, elderberry juice concentrate [53]	Inhibition of lipid peroxidation [21] Suppressing protein levels of NOX1 and NOX4 Up-regulating Nrf2 [86] ↓MDA [85]	↓ CRP ↓ IL-1β [82] Up-regulation of the PPARα ↓ Serum leptin and resistin ↓ TNF-α ↓ IL-6 ↓ IL-12 Suppressing iNOS and COX-2 Inhibition of signaling pathway of MAPK and NF-κB [86] ↓ MCP-1 [87]	↓ BMI ↓ Body weight [88] ↓ ApoB [82] ↑ HDL ↓ LDL ↓ Cholesterol ↓ TG ↓ Hypertrophy of the adipocytes in epididymal WAT [86]	↓ Insulin resistance GLUT4 and GLUT1, and thereby Improvement in insulin sensitivity	↓ Systolic blood pressure [53]	↑ Von Willebrand factor ↓ Endothelial dysfunction ↓ P-selectin [82] Inhibition of ICAM-1 and VCAM-1 ↑ eNOS expression and NO release [87]
**Genistein (Isoflavones)**	Soy and fava beans, green bean, kudzu root, and alfalfa sprouts [53]	Preventing phosphorylation of JNK Activation of Akt /ERK 1,2 pathway [89] ↑ eNOS activity and blocking NADPH-stimulated ROS production Suppression of superoxide production and NOX4 expression ↓ MDA [90]	↓ CRP [53] ↓ TNF-α [82] Activation of PPARs and AMPK ↓ IL-6 [89]	↓ TG ↓ Cholesterol ↓ Visfatin ↓ LDL and ox-LDL ↑ HDL ↑ Adiponectin [53] ↑ β-oxidation of FA ↓ Lipogenesis Preventing de-novo lipid synthesis [89] Promotion of browning of white adipocytes [90]	↓ Insulin resistance [82] ↑ Proliferation of βcells [89] ↓ HbA1c ↓ Blood glucose [90]	↓ Blood pressure [53]	↓ Circulating ICAM ↓ Endothelial dysfunction [53] ↓ ET-1 NO production via PKA/eNOS/NO signaling ↑ Expression of E-selectin, P-selectin, MCP-1, and IL-8 [90]
**Carotenoids**	Fruits (tangerines, cantaloupes, papayas, and oranges) and vegetables (carrot, pumpkin, spinach, sweet potato, tomato, broccoli, and green peas) [53]	↓ Production of free radicals [91] Serve as precursors for retinol (vitamin A), retinaldehyde, and retinoic acid, among other substances; retinoid conversion products that play important roles as transcriptional regulators in the visual cycle and gene regulation link [92]	Down-regulation of NF-κB and MAPK signaling pathways, Reducing the release of pro-inflammatory cytokines [93]	↑ Adiponectin ↓ Body weight ↓ Visceral fat ↓ Lipid storage ↓ LDL [53] ↓ TG ↑ HDL [91] Reversing cholesterol transport by HDL [93]	↑ Insulin sensitivity ↓ Insulin resistance [53] ↓ Blood glucose [91]	↓ Blood pressure [53]	↓ Endothelial dysfunction ↓ PAI-1 [53] Delay the progression of cardiovascular diseases ↑ NO bioavailability ↓ Accumulation of cholesterol in foam cells and the formation of atherosclerotic plaques [93]
**Resveratrol**	Grapes, apples, blueberries, plums, wine, peanuts [6], and dark chocolate [53]	↓ H_2_O_2_ production ↑ Level of regulatory T cells ↓ ROS by inhibiting the MAPK pathways [6]	Inhibition of PKA and Akt/PKB pathway [6]	Acting as a WAT remodeling to BAT ↓ Accumulation of glycerol in adipose tissue Promoting thermogenesis by activation of SIRT1 and suppressing white adipogenesis [6]	↑ Insulin production ↓ Insulin resistance [87] ↓ Blood glucose [94]	↓ Blood pressure ↑ Na^+^ excretion (renal) [53]	↑ NO production Up-regulation of eNOS expression Suppressing the synthesis of ET-1 [6]
**N-acetylcysteine**	Allium plant [87]	↑ Endogenous concentrations of total glutathione Scavenging free radicals Suppressing ROS generation [95] ↑ Intracellular cysteine levels Replenishing systemic pools of (LMW) thiols and reduced protein sulfhydryl groups, which are implicated in the regulation of the redox stats [96]	↓ CRP [97] Blocking NF-κB Inhibition of the release of IL-1, IL-6, and TNF [96] ↓ Gene expression of pro-inflammatory cytokines [33]	↓ TG ↑ HDL [97] ↓ Cholesterol ↓ LDL ↓ VLDL Preventing lipid accumulation in BAT [96]	↑ Insulin secretion [96] ↓ Blood glucose ↓ Insulin resistance [95]	↓ Blood pressure [87]	↑ NO [97] Stabilizing the production of atherosclerotic plaque [96]

NADPH oxidase (NOX), C-reactive protein (CRP), tumor necrosis factor (TNF), triglyceride (TG), low-density lipoprotein (LDL), oxidized low-density lipoprotein (ox-LDL), very low-density lipoprotein (VLDL), high-density lipoprotein (HDL), reactive oxygen species (ROS), interferon gamma (IFN-γ), nuclear factor erythroid 2–related factor 2 (Nrf2), endothelial NOS (eNOS), inducible nitric oxide synthase (iNOS), peroxisome proliferator-activated receptor α (PPAR-α), prostaglandin E2 (PGE2), plasminogen activator inhibitor-1 (PAI-1), intercellular adhesion molecule 1 (ICAM-1), vascular cell adhesion molecule 1(VCAM-1), glutathione (GSH), xanthine oxidase (XO), superoxide dismutase (SOD), glutathione peroxidase (GPx) glutathione reductase (GR), thioredoxin reductase (TrxR), catalase (CAT), triglyceride lipase (TGL), glycosylated hemoglobin (HbA1c), malondialdehyde (MDA), cyclooxygenase-2 (COX-2), free fatty acids (FFA), white adipose tissue (WAT), brown adipose tissue (BAT), monocyte chemoattractant protein 1 (MCP-1), apolipoprotein B (ApoB), endothelin 1 (ET-1), total antioxidant capacity (TAC), glucose transporters (GLUTs), body mass index (BMI), ↑: increase, ↓: decrease.

## Data Availability

We used PubMed and Web of Science to screen articles for this narrative review. We did not report any data.
